# Postoperative Complications Associated with Non-Steroidal Anti-Inflammatory Combinations Used Status-Post Total Hip and Knee Arthroplasty

**DOI:** 10.3390/jcm12226969

**Published:** 2023-11-07

**Authors:** Haley Nakata, Tara Shelby, Jennifer C. Wang, Gabriel J. Bouz, Cory K. Mayfield, Daniel A. Oakes, Jay R. Lieberman, Alexander B. Christ, Nathanael D. Heckmann

**Affiliations:** Department of Orthopaedic Surgery, Keck School of Medicine of USC, Los Angeles, CA 90033, USA; haley.nakata@med.usc.edu (H.N.); tshelby@usc.edu (T.S.); jennifer.wang@hsc.utah.edu (J.C.W.); gabriel.bouz@med.usc.edu (G.J.B.); cory.mayfield@med.usc.edu (C.K.M.); daniel.oakes@med.usc.edu (D.A.O.); jay.lieberman@med.usc.edu (J.R.L.); alexander.b.christ@gmail.com (A.B.C.)

**Keywords:** total joint arthroplasty, non-steroidal anti-inflammatory drugs, acute kidney injury, stroke

## Abstract

Non-steroidal anti-inflammatory drugs (NSAIDs) are commonly used in multimodal pain control following total joint arthroplasty (TJA). However, few studies have assessed the complications associated with the combinations of NSAIDs in this population despite the known risks associated with this class of medications. The Premier Healthcare Database was queried to identify adults who underwent primary total hip or knee arthroplasty from 2005–2014. The following most common inpatient combinations of NSAIDs were chosen for analysis: aspirin + celecoxib (A + C), toradol + aspirin (T + A), toradol + ibuprofen (T + I), celecoxib + ibuprofen (C + I), ibuprofen + aspirin (I + A), and toradol + celecoxib (T + C). Primary outcomes included acute kidney injury (AKI), gastrointestinal bleed, and stroke. Secondary outcomes included periprosthetic joint infection (PJI), deep vein thrombosis, and pulmonary embolism. Univariate and multivariate regression analyses were used to compare differences and address confounds. Overall, 195,833 patients were identified. After controlling for confounds, increased odds of AKI was associated with A + C (adjusted odds ratio [aOR]: 1.20, 95% confidence interval [CI]: 1.09–1.34, *p* < 0.001) and decreased odds was associated with T + A (aOR 0.76, 95% CI: 0.69–0.83, *p* < 0.001). Increased odds of stroke was associated with A + C (aOR: 1.80, 95% CI: 1.15–2.84, *p* = 0.011); T + I (aOR 3.48, 95% CI: 1.25–9.73, *p* = 0.017); and I + A (aOR 4.29, 95% CI: 1.06–17.9, *p* = 0.046). Increased odds of PJI was associated with C + I (aOR: 10.3, 95% CI: 1.35–78.3, *p* = 0.024). In the TJA patient population, NSAID pairings should be regarded as distinct entities. Our results suggest that combinations including A + C, T + I, I + A, and C + I should be used cautiously. With this knowledge, providers should consider tailoring NSAID prescriptions appropriately.

## 1. Introduction

Increased attention has been drawn to multimodal analgesia protocols that use non-opioid pharmacotherapy, such as non-steroidal anti-inflammatory drugs (NSAIDs), to decrease perioperative opioid consumption. Multiple studies have noted reduced opioid consumption and fewer adverse events when modern multimodal pain regimens are employed [1,2,3,4,5]. The etiology of postoperative pain associated with both total hip (THA) and total knee arthroplasty (TKA) is multifactorial and related to damage to cells, which results in chemical mediator release and the sensitization of nociceptors [6]. The medications used in modern multimodal analgesia protocols target many of the molecular pathways involved in producing these biomolecules.

The antinociceptive effects of NSAIDs are derived from the inhibition of prostaglandin synthase G/H, also known as cyclooxygenase (COX) 1 and 2 [7]. COX-1 is constitutively expressed in all cells whereas COX-2 is expressed at sites of tissue injury [8]. COX-1 enzymes regulate gastrointestinal (GI) cytoprotectin, platelet function, and renal function, as well as produce thromboxane and prostacyclin [8]. However, the biologic pathway by which NSAIDs produce pain relief also gives rise to the known side effects of this class of medications. These include acute kidney injury (AKI), GI bleeding, and thrombotic events such as stroke. NSAIDs can cause GI side effects through COX-1 inhibition and atherothrombotic vascular events through COX-2 inhibition [9,10]. The increased prothrombotic risk of COX-2-selective NSAIDs may be attributed to a disproportionate decrease in prostacyclin, which is a vasodilator, platelet inhibitor, and relative to thromboxane [8]. In terms of kidney damage, the NSAID inhibition of COX enzymes centrally and peripherally interferes with the conversion of arachidonic acid into prostaglandins, prostacyclins, and thromboxanes [11]. In turn, the vasodilatory effects of prostaglandins are inhibited, compromising glomerular filtration rate and renal blood flow.

Currently, there is a paucity in the literature regarding characterizing the complications associated with combined NSAID usage, which is an increasingly common practice in modern analgesia pathways [12]. Multiple studies have looked at NSAID use and the association with AKI, GI bleeds, and strokes following THA and TKA [13,14,15,16]. However, these studies were limited by a focus on a single NSAID, or by grouping all NSAIDs together without parsing out the specific risks associated with each combination [13,17,18]. A recently published single-institution study examined the renal complications of the combined use of ibuprofen with aspirin, but it did not investigate other postoperative complications nor other combinations of NSAIDs [19]. Therefore, the purpose of this study was to determine the adverse effects of commonly used inpatient NSAID combinations, and to—in particular—determine which specific combinations demonstrate the highest risk profiles regarding AKI, GI bleeds, and atherothrombotic events.

## 2. Materials and Methods

### 2.1. Data Source and Population

This was a retrospective cohort study that was conducted using the Premier Healthcare Database (PHD) (Premier, Inc., Charlotte, NC, USA). The PHD is a United States (U.S.) hospital-based, comprehensive electronic healthcare database with contributions from over 700 U.S. hospitals and health care systems in 2014 [20]. De-identified data pertaining to patient demographics, disease status, procedure type, and hospital characteristics are available through the PHD, as well as information pertaining to medications administered during patient visits.

This study was exempt from the consideration of an institutional review board review as all patient information was de-identified in accordance with the Health Insurance Portability and Accountability Act.

### 2.2. Identification of Study Cohorts

International Classification of Diseases 9th edition (ICD-9) codes 81.51 and 81.54 were utilized to identify adults who underwent primary elective THA or TKA from 2005 to 2014. Patients who were <18 years old and who underwent TJA for non-elective indications were excluded (Section S1). Billing codes for NSAIDs were then used to identify the most common NSAIDs used from 2005–2014 (Supplementary Table S1). The six most common combinations were toradol and celecoxib (T + C), aspirin and celecoxib (A + C), toradol and aspirin (T + A), toradol and ibuprofen (T + I), celecoxib and ibuprofen (C + I), and ibuprofen and aspirin (I + A). Patients receiving one of these six combinations comprised the study cohorts. NSAID totals were calculated based on postoperative day 0 (POD0) orders to account for the potential confounders of (1) physicians recommending NSAID cessation in select patients secondary to the development of complications later in the postoperative course and (2) variability in length of stay.

### 2.3. Statistical Analysis

Descriptive statistics were utilized to summarize patient and hospital characteristics of each cohort. The given observation number, skewness, and the kurtosis test for normality were chosen to assess the normality of the variables. Differences between demographic variables were determined using a chi-square for categorical variables, and an analysis of variance was conducted for continuous variables. Logistic regression was utilized to determine the relationships between each NSAID combination and postoperative outcomes. Primary outcomes included AKI, GI bleeds, and stroke. Secondary outcomes included periprosthetic joint infection (PJI), deep vein thrombosis (DVT), pulmonary embolism (PE), readmission, and hematoma. All outcomes examined occurred within a 90-day postoperative timeframe. Odds ratios (OR) were calculated for each combination with T + C, as the most common combination, serving as the reference group.

Three models were used for a multivariate regression to determine the association between NSAID combination and each of the primary and secondary outcomes. Model 1 controlled for age, race, and gender. Model 2 controlled for age, race, gender, and significant comorbidities (as determined by univariate analyses). Model 3 controlled for age, race, gender, significant comorbidities, as well as hospital region, teaching status, and urban/rural status. For each regression model, poorly predictive variables were omitted from the independent variable list, and a variance inflation factor was used to detect multicollinearity.

An additional sensitivity analysis was conducted to identify the patient comorbidities associated with each primary and secondary endpoint. Adjusted odds ratios (aOR) for each comorbidity were calculated by controlling for all other included comorbidities.

All statistical analyses were performed using STATA (version 13.0; StataCorp LLC, College Station, TX, USA).

## 3. Results

### 3.1. Trends in Type of NSAID Ordered

**Figure 1:**
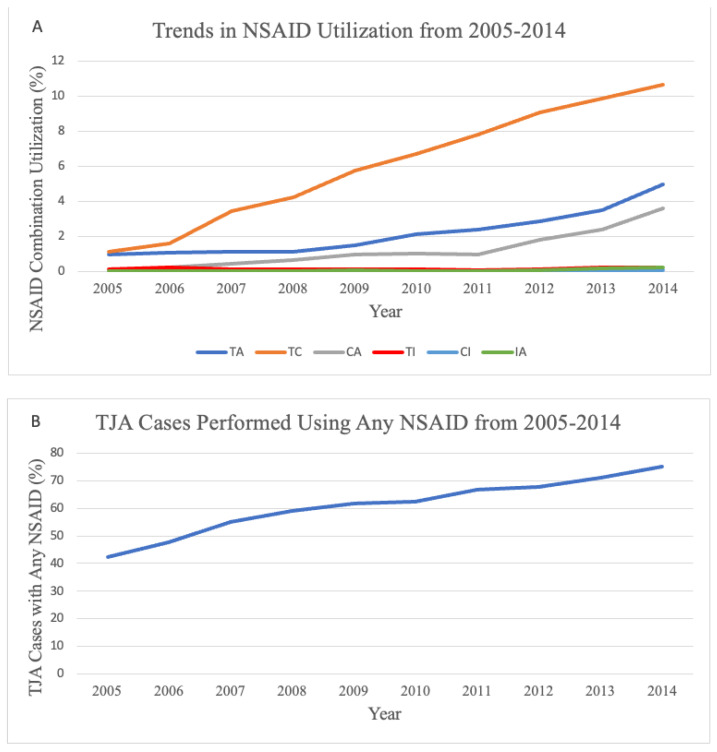
Changes in the prevalence of NSAID usage, represented as a percentage of total joint arthroplasty (TJA) cases performed annually, from 2005–2014. (**A**) Percentage utilization of various NSAID combinations. (**B**) Percentage of TJA cases performed in which any NSAID was utilized. NSAID: nonsteroidal anti-inflammatory drug; TC: toradol + celecoxib; TA: toradol + aspirin; CA: celecoxib + aspirin; TI: toradol + ibuprofen; CI: celecoxib + ibuprofen; and IA: ibuprofen + aspirin.

Between 2005 and 2014, there were 1,702,591 elective primary TJAs performed (69.4% TKA, 30.6% THA). The proportion of cases utilizing at least one perioperative NSAID increased by 32.6% (2005: 42.3%, 2014: 75.1%) while the proportion of cases using any combination of NSAIDs increased by 9.17% (2005: 2.3%, 2014: 19.7%) (Figure 1). During this timeframe, the combinations of T + C (mean: 6.02%), T + A (mean: 2.16%) and C + A (mean: 1.21%) persisted as the most common combinations. The mean prevalence of the remaining NSAID combinations—T + I, C + I, and I + A—was smaller at 0.14%, 0.02%, and 0.06%, respectively.

### 3.2. Patient and Hospital Demographics

There were 195,833 patients identified who met the inclusion criteria for further analysis (Table 1). Of these, 62.0% (121,393) received a combination of T + C; 13.5% (26,345) received A + C; 22.4% (43,854) received T + A; 1.3% (2547) received T + I; 0.2% (395) received C + I; and 0.7% (1299) received I + A. Between the six treatment groups, there were statistically significant differences in sex, race, length of stay, patient cost, insurance type, geographic region, hospital teaching status, and rural versus urban hospital location (*p* < 0.001) (Table 1). Statistically significant differences in age were present but were not clinically meaningful (range: 64.6–65.8 years).

### 3.3. Patient Comorbidities

Statistically significant differences in the prevalence of hypertension (*p* < 0.001), renal failure (*p* < 0.001), and vascular disease (*p* < 0.001) were identified across the cohorts (Table 2). There were no differences in the prevalence of diabetes, HIV/AIDS, or congestive heart failure. CHF = congestive heart failure; PUD = peptic ulcer disease.

Increased risk of AKI was associated with multiple comorbidities, including CHF (OR: 9.76, 95% CI: 9.32–10.21, *p* < 0.001), complicated diabetes (OR: 9.24, 95% CI: 9.72–9.80, *p* < 0.001), and renal failure (OR: 24.8, 95% CI: 23.9–25.6, *p* < 0.001) (Supplementary Table S2). Increased risk of stroke was associated with HIV/AIDS (OR: 5.66, 95% CI: 4.55–7.00, *p* < 0.001), pulmonary vascular disease (OR: 5.60, 95% CI: 4.50–7.10, *p* < 0.001), and pulmonary circulation disorders (OR: 7.60, 95% CI: 5.60–10.2, *p* < 0.001), amongst other comorbidities (Supplementary Table S2). Similarly, increased risk of GI bleeding was also associated with comorbidities including HIV/AIDS (OR: 7.80, 95% CI: 5.90–10.4, *p* < 0.001), pulmonary vascular disease (OR: 6.40, 95% CI: 4.60–8.90, *p* < 0.001), and pulmonary circulation disorders (OR: 10.0, 95% CI: 6.80–14.9, *p* < 0.001) (Supplementary Table S2).

### 3.4. Primary Endpoints: Acute Kidney Injury, Stroke, and Gastrointestinal Bleeding

Across all treatment groups, the prevalence of AKI was between 0.09–2.53%, the highest of which was in the C + I cohort and the lowest in the I + A cohort (Table 3). After controlling for confounders, the A + C cohort was associated with increased odds (aOR 1.20, *p* < 0.001) and the T + A cohort was associated with decreased odds of AKI (aOR 0.76, *p* < 0.001).

The prevalence of stroke across the cohorts ranged from 0.05–0.25%, the greatest of which was seen in the C + I cohort and the lowest of which in the T + C cohort. After controlling for confounders, the A + C, T + I, and I + A cohorts were all associated with increased odds of stroke (A + C: aOR 1.80, *p* = 0.011; T + I: aOR 3.48, *p* = 0.017; I + A: aOR 4.29, *p* = 0.046).

The prevalence of GI bleeding was between 0.00–6.86% across the six cohorts, with no cases seen in the T + I, C + I, and I + A cohorts, and the highest number of cases seen in the T + C cohort. The odds of GI bleeding was lower in the T + A cohort compared to the T + C reference; however, this difference was not statistically significant (aOR 0.47, *p* = 0.144). Odds ratios for the other treatment cohorts could not be computed due to prohibitive sampling sizes or the absence of cases.

In summary, the A + C combination was associated with increased odds of AKI and stroke, the T + A combination was associated with decreased odds of AKI, and the T + I combination was associated with an increased odds of stroke across all multivariate models. The I + A combination was associated with an increased odds of stroke in Model 3. There were no significant differences noted in GI bleeding rates between cohorts.

No multi-collinearity was detected across the different primary outcomes, and all VIF values were less than 5.

### 3.5. Secondary Endpoints: Other Postoperative Complications

Across all of the six treatment combinations, the prevalence of PJI was between 0.02–0.25%, the highest of which was in the C + I cohort (0.25%). However, the prevalence of PJI was equally low in the T + C, A + C, and T + A cohorts (0.02%) (Table 4). In the third multivariate model, an increased risk of PJI was associated with C + I (aOR: 10.3, 95%-CI: 1.35–78.3, *p* = 0.024).

The prevalence of DVT ranged from 0.00–0.19% amongst the cohorts, the highest of which was in the T + C and T + A cohorts (0.19%). No DVTs were noted in the C + I and I + A cohorts (Table 4). In the third multivariate model, a decreased risk of DVT was associated with the T + A cohort (aOR: 0.76, 95%-CI: 0.60–0.98, *p* = 0.039).

The prevalence of PE ranged from 0.00–0.25% amongst the cohorts, the highest of which was in the C + I cohort (0.25%) and lowest in the I + A cohort (0.00%) (Table 4). No significant associations between the development of PE and treatment combination were identified.

The readmission rate was 3.64–4.81% amongst the cohorts, the highest of which was in the C + I cohort (4.81%) and lowest in the A + C cohort (3.64%) (Table 4). In the third multivariate model, a decreased risk of readmission was associated with the A + C (aOR: 0.71, 95%-CI: 0.67–0.76, *p* < 0.001) and T + A cohorts (aOR: 0.71, 95%-CI: 0.68–0.75, *p* < 0.001).

The hematoma rate was 0.00–0.13% amongst the cohorts, the highest of which was in the T + C cohort (0.13%). There were no hematomas in the C + I and I + A cohorts (0.00%) (Table 4). In the third multivariate model, a decreased risk of hematoma formation was associated with the A + C (aOR: 0.50, 95%-CI: 0.31–0.81, *p* = 0.005) and T + A cohorts (aOR: 0.63, 95%-CI: 0.45–0.88, *p* = 0.007).

No multi-collinearity was detected across the different secondary outcomes, and all VIF values were less than 5.

## 4. Discussion

This study addresses an important gap in the literature by being the first to examine the safety profile of commonly used NSAID pairings following primary TKA and THA. This is a topic of importance as their use continues to increase in modern multimodal perioperative pain regimens. Each unique NSAID pairing was associated with a slightly different postoperative complication profile. Therefore, caution should be exercised when prescribing certain combinations, particularly in high-risk patients. Increased risk of AKI was associated with A + C; increased risk of stroke was observed in patients who received A + C, T + I, or I + A; and increased risk of PJI was associated with C + I. In contrast, patients who received T + A were at decreased risk of AKI, DVT, postoperative hematoma, and unplanned readmission. These results demonstrate that NSAID pairings should be regarded as separate and distinct entities, as well as ultimately suggest that A + C, T + I, I + A, and C + I should be used with caution in the TJA patient population.

There are several studies that investigate the risk of AKI associated with NSAID utilization. The effect of NSAIDs on kidney function is mediated by COX-1 inhibition, thereby explaining the theoretical advantage of COX-2-selective inhibitors [8]. The adverse renal side effects from these medications may be even more deleterious in the postoperative period as patients may be volume depleted and thus more vulnerable to kidney injury. Multiple studies have corroborated the hypothesis that COX-2-selective NSAIDs are associated with a lower risk of AKI [18,21,22,23,24,25]. However, our results expand on previous findings by demonstrating that aspirin in combination with celecoxib (A + C), a COX-2-selective NSAID, may be associated with an increased risk of AKI whereas combinations such as T + A may be associated with decreased risk of postoperative AKI.

The increased risk of stroke with COX-2-selective inhibitors is also well recognized [17,26]. The increased prothrombotic risk seen with these medications arises from the disruption of the prostacyclin and thromboxane balance [8]. In the present study, no combination of NSAIDs examined was associated with a decreased risk of stroke. Rather, there were three combinations (i.e., A + C, T + I, and I + A) seen to increase the risk of stroke. As such, use of these combinations should be implemented with caution, particularly in high-risk patients. Interestingly, even though aspirin is often used for VTE prophylaxis, as it is an inhibitor of COX-1 and platelet aggregation [27,28,29,30,31], there were no combinations using aspirin that were associated with a protective effect against stroke.

In contrast to the data on AKI and strokes, there is a lack of consensus in the prior literature regarding the examining of postoperative GI bleeding associated with NSAID use. Fleischmann et al. found no association with non-aspirin NSAID use but found an increased risk of GI bleeding with aspirin; in anticoagulants such as warfarin; direct oral anticoagulants; and antiplatelet agents [13]. Several studies have suggested a preoperative GI diagnosis of reflux or peptic ulcer disease is associated with an increased risk of postoperative GI bleeding; however, aspirin is not associated with an increased risk of GI bleeding, irrespective of preoperative GI diagnosis [14,15,16,32]. Though the risk of GI bleeding was presently found to be lower in the T + A cohort compared to the T + C cohort, the results pertaining to GI bleeding in our study were limited by sample size and case prevalence. This underscores the need for further investigation into this topic with larger cohorts to more accurately characterize the risk profile of NSAIDs, which is increasingly important as they are increasingly used in modern arthroplasty practice settings.

The present study has several limitations. First, the findings rely on the accuracy of ICD-9 codes, which are subject to errors. However, such an error, if present, would likely not preferentially affect one treatment cohort over another. Furthermore, the study is limited to ICD-9 codes and the associated data constraints prior to switching to ICD-10 coding. Using both ICD-9 and ICD-10 codes would not allow for accurate data collection [33]. The retrospective nature of this study also holds the assumption that if a complication occurred, it would be captured through the coding system. Second, the retrospective nature of this study introduces the possibility of unidentified confounders, such as uncoded patient or surgical factors, which may have influenced a surgeon to select one NSAID combination over another. However, all identifiable confounders were accounted for through three carefully constructed multivariate models of increasing complexity. Third, the present study only examined a 90-day follow-up period, thereby preventing the capture of longer-term complications. However, we expect our three primary endpoints to occur in the early postoperative period and, as such, would not expect our findings to change markedly with longer follow-up. Concurrently, due to the prohibitively small number of cases of GI bleeding, our multivariate model was unable to produce meaningful results for this endpoint. Lastly, the duration of usage for each NSAID following discharge was not available in the PHD, and it likely varied based on common practice patterns. For example, toradol is often administered intravenously for a shorter duration than celecoxib and meloxicam, which are often given for extended durations postoperatively [34]. However, the focus of this study was to assess the risk profile of NSAID combinations in the way they are commonly used. As such, the variability of duration of use in this large sampling of patients is a strength as this represents the common utilization practices amongst orthopaedic surgeons. Further limitation of the present study involves not accounting for drug interactions, pharmacokinetics, and pharmodynamics, as well as the possible effects of drug dosage and patient body weight.

Despite these limitations, this study provides valuable and novel insights into the relationships between commonly used NSAID combinations and the associated complications following TJA. This study included a large cohort of nearly 200,000 patients across many insurance types and practice settings, reinforcing the external validity of the study. In combination with this study’s robust statistical power, it is also the largest study to include the specific combinations of NSAIDs utilized. Previous studies have looked at the relationship between specific NSAIDs and various postoperative complications, but none to our knowledge have compared common NSAID combinations and their respective safety profiles.

## 5. Conclusions

Care should be taken when prescribing certain combinations of NSAIDs in the TJA patient population. Ultimately, the findings of this study suggest that combinations including A + C, T + I, I + A, and C + I should be prescribed with caution in the TJA patient population. With this knowledge, providers should consider the risks and benefits of certain combinations of NSAIDs in this population.

## Figures and Tables

**Table 1:** Differences in patient and hospital demographics.

	T + C		A + C		T + A		T + I		C + I		I + A		*p*-Value
Total Patients (*n*)	121,393 (62.0%)		26,345 (13.5%)		43,854 (22.4%)		2547 (1.3%)		395 (0.2%)		1299 (0.7%)		
Age (years)	65.04 ± 0.03		65.44 ± 0.08		65.79 ± 0.05		65.69 ± 0.26		64.57 ± 0.53		64.83 ± 0.28		<0.001
Length of Stay (days)	2.66 ± 0.00		2.48 ± 0.01		2.86 ± 0.01		2.82 ± 0.04		2.96 ± 0.17		1.81 ± 0.04		<0.001
Cost of Hospitalization (USD)	16,053.97 ± 20.19		15,237.62 ± 42.18		15,436.36 ± 55.00		15,688.10 ± 148.28		16,445.46 ± 573.6		13,884.32 ± 112.62		<0.001
	*n*	%	*n*	%	*n*	%	*n*	%	*n*	%	*n*	%	
Sex													<0.001
Female	68,462	56.4	10,645	40.4	24,772	56.5	1093	42.9	210	53.2	718	55.3	
Race/Ethnicity													<0.001
White	99,667	82.1	22,220	84.34	36,247	82.65	1770	69.49	306	77.47	687	52.9	
Black	8330	6.9	2047	7.77	3227	7.36	122	4.79	31	7.85	69	5.3	
Hispanic	909	0.8	92	0.35	109	0.25	9	0.35	0	0.00	3	0.2	
Other	12,304	10.1	1916	7.27	4134	9.43	644	25.28	58	14.68	538	41.4	
Unspecified	183	0.2	70	0.27	137	0.31	2	0.08	0	0.00	2	0.2	
Insurance Type													<0.0021
Medicare	62,552	51.5	9999	52.2	12,106	57.5	1431	56.18	200	50.63	727	56.0	
Medicaid	3407	2.8	600	2.6	545	2.6	84	3.30	17	4.30	42	3.2	
Managed Care	41,060	33.8	6637	33.1	5957	28.3	791	31.06	136	34.43	416	32.0	
Private	9258	7.6	1441	7.5	1454	6.9	118	4.63	30	7.59	48	3.7	
Other	5116	4.2	982	4.9	982	4.7	123	4.83	12	3.04	66	5.1	
Geographic Region													<0.001
Midwest	25,997	21.4	4640	17.61	9456	21.56	257	10.09	108	27.3	39	3.0	
Northeast	15,267	12.6	5594	21.23	6479	14.77	615	24.15	59	14.9	102	7.9	
South	53,031	43.7	11,582	43.96	21,787	49.68	1408	55.28	129	32.7	1124	86.5	
West	27,098	22.3	4529	17.19	6132	13.98	267	10.48	99	25.1	34	2.6	
Teaching Status													<0.001
Teaching	49,983	41.2	11,683	44.3	18,724	42.7	355	13.9	219	55.4	150	11.5	
Urban vs. Rural													<0.001
Rural	13,077	10.8	1919	7.3	4593	10.5	286	11.2	74	18.7	29	2.2	

**Table 2:** Patient comorbidities by NSAID group.

	T + C		A + C		T + A		T + I		C + I		I + A		*p*-Value
	*n*	%	*n*	%	*n*	%	*n*	%	*n*	%	*n*	%	
Diabetes	1209	1.00	264	1.00	603	1.38	16	0.63	4	1.01	9	0.69	0.521
Hypertension	61,972	51.1	12,127	46.0	28,324	64.6	1105	43.4	222	56.2	692	53.3	**<0.001**
Renal Failure	3059	2.52	870	3.30	1613	3.68	32	1.26	12	3.04	25	1.92	**<0.001**
Valvular Disease	3320	2.73	732	2.78	1670	3.81	69	2.71	9	2.28	17	1.31	**<0.001**
HIV/AIDS	36	0.03	2	0.01	28	0.06	0	0.00	0	0.00	1	0.08	0.198
CHF	1895	1.56	376	1.43	953	2.17	32	1.26	14	3.54	25	1.92	0.096
Hypothyroidism	6828	5.62	17,966	68.2	3978	9.07	341	13.4	63	15.9	118	9.08	**<0.001**
Liver Disease	353	0.29	1041	3.95	230	0.52	23	0.90	2	0.51	6	0.46	**<0.001**
Peripheral Vascular Disease	914	0.75	2242	8.51	562	1.28	35	1.37	5	1.27	23	1.77	**<0.001**
Pulmonary Circulation Disorders	337	0.28	787	2.99	168	0.38	16	0.63	5	1.27	4	0.31	**<0.001**
Chronic PUD	3	0.00	12	0.05	7	0.02	0	0.00	0	0.00	0	0.00	0.531
Chronic Pulmonary Disease	6194	5.10	16,241	61.6	3456	7.88	319	12.5	54	13.7	147	11.3	**<0.001**

Bold values are statistically significant (*p* < 0.001). HIV = human immunodeficiency virus, AIDS = acquired immunodeficiency syndrome.

**Table 3:** Univariate and multivariate analyses that were conducted to examine the risk of developing acute kidney injury (AKI), stroke, and gastrointestinal bleeding (GIB) within 90 days of admission amongst the six cohorts. T + C: toradol with celecoxib, A + C: aspirin with celecoxib, T + A: toradol with aspirin, T + I: toradol with ibuprofen, C + I: celecoxib with ibuprofen, and I + A: ibuprofen with aspirin. / = no value can be obtained; AKI = acute kidney injury; and GIB = gastrointestinal bleeding.

				Univariate Analysis			Model 1			Model 2			Model 3		
Complication	Combination	*n*	%	OR	CI	*p*-Value	aOR	CI	*p*-Value	aOR	CI	*p*-Value	aOR	CI	*p*-Value
AKI	T + C	1664	1.37%	Ref	Ref		Ref	Ref		Ref	Ref		Ref	Ref	
	A + C	476	1.81%	1.32	1.19–1.47	<0.001	1.22	1.1–1.36	0.000	1.08	0.97–1.20	0.000	1.20	1.09–1.34	0
	T + A	608	1.39%	0.82	0.75–0.91	<0.001	0.81	0.73–0.88	0.000	0.75	0.68–0.83	0.000	0.76	0.69–0.83	0
	T + I	28	1.10%	0.79	0.55–1.16	0.24	0.75	0.52–1.10	0.140	0.80	0.55–1.17	0.247	0.78	0.53–1.14	0.195
	C + I	10	2.53%	1.87	0.99–3.50	0.05	1.90	1.01–3.58	0.047	1.71	0.90–3.24	0.100	1.69	0.89–3.2	0.109
	I + A	15	0.09%	0.84	0.50–1.40	0.505	0.76	0.45–1.26	0.286	0.95	0.56–1.59	0.833	0.93	0.55–1.57	0.786
Stroke	T + C	62	0.05%	Ref	Ref		Ref	Ref		Ref	Ref		Ref	Ref	
	A + C	27	0.10%	2.01	1.28–3.16	0.015	1.80	1.15–2.83	0.011	1.71	1.08–2.69	0.021	1.80	1.15–2.84	0.011
	T + A	34	0.08%	1.51	1.00–2.31	0.280	1.18	0.77–1.79	0.440	1.14	0.75–1.73	0.500	1.15	0.76–1.76	0.500
	T + I	4	0.16%	3.07	1.12–8.45	0.030	3.00	1.09–8.26	0.034	3.09	1.12–8.51	0.030	3.48	1.25–9.73	0.017
	C + I	1	0.25%	4.97	0.69–35.9	0.140	5.24	0.72–37.98	0.101	4.71	0.65–34.34	0.126	4.82	0.66–35.14	0.121
	I + A	3	0.22%	4.53	1.42–14.5	0.010	3.31	0.80–13.65	0.098	3.93	0.95–16.26	0.050	4.29	1.06–17.89	0.046
GIB	T + C	31	6.86%	Ref	Ref		Ref	Ref		Ref	Ref		Ref	Ref	
	A + C	2	0.44%	/	/	0.040	/	/	/	/	/	/	/	/	/
	T + A	8	1.77%	0.71	0.33–1.55	0.182	0.46	0.14–1.51	0.201	0.47	0.14–1.55	0.216	0.47	1.56–220.0	0.144
	T + I	0	0.00%	/	/	0.390	/	/	/	/	/	/	/	/	/
	C + I	0	0.00%	/	/	0.735	/	/	/	/	/	/	/	/	/
	I + A	0	0.00%	/	/	0.540	/	/	/	/	/	/	/	/	/

**Table 4:** Univariate and multivariate analyses conducted to examine the risk of developing secondary outcomes of interest within 90 days of admission amongst the six cohorts. T + C: toradol with celecoxib, A + C: aspirin with celecoxib, T + A: toradol with aspirin, T + I: toradol with ibuprofen, C + I: celecoxib with ibuprofen, and I + A: ibuprofen with aspirin. PJI: prosthetic joint infection, DVT = deep vein thrombosis, and PE = pulmonary embolism.

Complication	Combination	Univariate					Model 1			Model 2			Model 3		
		*n*	% of NSAID Group	OR	CI	*p*-Value	aOR	CI	*p*-Value	aOR	CI	*p*-Value	aOR	CI	*p*-Value
PJI	T + C	23	0.02	Reference											
	A + C	6	0.02	1.2	0.50–2.95	0.690	1.11	0.45–2.70	0.816	1.10	0.40–2.70	0.840	1.20	0.49–2.90	0.700
	T + A	9	0.02	1.08	0.50–2.30	0.839	0.89	0.40–1.90	0.700	0.87	0.40–1.90	0.700	0.90	0.40–1.90	0.750
	T + I	1	0.04	2.07	0.30–15.4	0.482	2.02	0.27–15.0	0.492	2.18	0.30–16.0	0.445	2.30	0.30–17.4	0.419
	C + I	1	0.25	13.4	1.80–99.3	**0.011**	13.8	1.90–102.6	**0.010**	12.1	1.60–91.6	**0.015**	10.3	1.35–78.3	**0.024**
	I + A	1	0.08	4.1	0.55–30.1	0.170	3.56	0.50–26.6	0.216	4.00	0.53–30.1	0.177	5.50	0.71–42.0	0.103
DVT	T + C	235	0.19	Reference											
	A + C	46	0.17	0.90	0.66–1.24	0.520	0.84	0.60–1.15	0.294	0.92	0.60–1.13	0.242	0.80	0.60–1.1	0.220
	T + A	83	0.19	0.98	0.76–1.30	0.177	0.79	0.61–1.01	0.066	0.78	0.60–1.00	0.052	0.76	0.60–0.98	**0.039**
	T + I	2	0.08	0.40	0.10–1.63	0.202	0.40	0.100–1.60	0.192	0.41	0.10–1.66	0.212	0.43	0.11–1.74	0.237
	C + I	0	0.00	-	-	-	-	-	-	-	-	-	-	-	-
	I + A	0	0.00	-	-	-	-	-	-	-	-	-	-	-	-
PE	T + C	203	0.17	Reference											
	A + C	58	0.22	1.30	0.98–1.76	0.065	1.23	0.92–1.64	0.167	1.21	0.90–1.62	0.200	1.20	0.90–1.60	0.232
	T + A	107	0.24	1.46	1.16–1.85	**0.002**	1.16	0.92–1.47	0.196	1.15	0.91–1.46	0.233	1.13	0.90–1.43	0.300
	T + I	2	0.08	0.47	0.11–1.88	0.285	0.45	0.11–1.80	0.260	0.47	0.12–1.88	0.283	0.47	0.12–1.88	0.285
	C + I	1	0.25	1.50	0.21–10.8	0.680	1.50	0.21–10.8	0.680	1.40	0.20–10.2	0.728	1.35	0.19–9.70	0.763
	I + A	0	0.00	-	-	-	-	-	-	-	-	-	-	-	-
Readmission	T + C	5623	4.63	Reference											
	A + C	960	3.64	0.78	0.73–0.83	**<0.001**	0.73	0.68–0.78	**<0.001**	0.72	0.67–0.77	0.000	0.71	0.67–0.76	**<0.001**
	T + A	1824	4.16	0.89	0.85–0.94	**<0.001**	0.73	0.69–0.77	**<0.001**	0.72	0.68–0.76	0.000	0.71	0.68–0.75	**<0.001**
	T + I	101	3.97	0.85	0.70–1.04	0.109	0.83	0.68–1.02	0.074	0.85	0.70–1.05	0.127	0.97	0.79–1.18	0.743
	C + I	19	4.81	1.04	0.66–1.65	0.870	1.05	0.66–1.67	0.834	1.03	0.64–1.63	0.916	1.00	0.63–1.6	0.993
	I + A	50	3.85	0.82	0.62–1.09	0.181	0.81	0.60–1.07	0.143	0.85	0.64–1.12	0.250	0.96	0.70–1.27	0.762
Hematoma	T + C	162	0.13	Reference											
	A + C	19	0.07	0.54	0.34–0.87	**0.005**	0.51	0.31–0.81	**0.005**	0.50	0.31–0.81	**0.005**	0.50	0.31–0.81	**0.005**
	T + A	45	0.10	0.77	0.55–1.07	0.119	0.63	0.45–0.88	**0.006**	0.63	0.45–0.87	**0.006**	0.63	0.45–0.88	**0.007**
	T + I	1	0.04	0.30	0.04–2.10	0.221	0.29	0.04–2.05	0.213	0.30	0.04–2.07	0.216	0.30	0.42–2.17	0.235
	C + I	0	0.00	-	-	-	-	-	-	-	-	-	-	-	-
	I + A	0	0.00	-	-	-	-	-	-	-	-	-	-	-	-

Bolded values are statistically significant (*p* < 0.05).

## Data Availability

The data presented in this study are available in the Premier Healthcare Database.

## Supplementary Materials

The following supporting information can be downloaded at: https://www.mdpi.com/article/10.3390/jcm12226969/s1, Table S1: Billing codes used to identify non-steroidal anti-inflammatory (NSAID) medications ordered for patients from 2005–2014; Table S2: Comorbidity prevalence in those with and without AKI, stroke, and GIB; Section S1: Criteria used to define non-elective cases for exclusion.
