# Supplementary material for: Postoperative Complications Associated with Non-Steroidal Anti-Inflammatory Combinations Used Status-Post Total Hip and Knee Arthroplasty
Source: J Clin Med. 2023 Nov 7;12(22):6969. doi: 10.3390/jcm12226969 (PMC10672686; doi:10.3390/jcm12226969)
Supplement: Supplementary file 1 [file jcm-12-06969-s001.zip › jcm-2661770-supplementary.pdf]

Postoperative Complications Associated with Non-Steroidal Anti-Inflammatory Combinations Used Status-Post Total Hip and Knee Arthroplasty

Haley Nakata, Tara Shelby, Jennifer C. Wang, Gabriel J. Bouz, Cory K. Mayfield, Daniel A. Oakes, Jay R. Lieberman, Alexander B. Christ and Nathanael D. Heckmann *

Department of Orthopaedic Surgery, Keck School of Medicine of USC, Los Angeles, CA 90033, USA; haley.nakata@med.usc.edu (H.N.); tshelby@usc.edu (T.S.); jennifer.wang@hsc.utah.edu (J.C.W.); gabriel.bouz@med.usc.edu (G.J.B.); cory.mayfield@med.usc.edu (C.K.M.); daniel.oakes@med.usc.edu (D.A.O.); jay.lieberman@med.usc.edu (J.R.L.); alexander.b.christ@gmail.com (A.B.C.)

* Correspondence: nate.heckmann@gmail.com

Table S1. Billing codes used to identify non-steroidal anti-inflammatory (NSAID) medications ordered for patients from 2005-2014.

Drug Name	Charge Code	Description
Aspirin	250250004400000	ASPIRIN TAB EC 975MG
	250250004430000	ASPIRIN TAB SR 800MG
	250250004450000	ASPIRIN/CAFF/BUT, FIORINAL CAP 325/40/50MG
	250250004460000	ASPIRIN/CAFF/BUT, FIORINAL TAB 325/40/50MG
	250250004480000	ASPIRIN/COD, EMPIRIN/COD #3 TAB 30/325MG
	250250004490000	ASPIRIN/COD, EMPIRIN/COD #4 TAB 60/325MG
	250257004260000	ASPIRIN TAB BUFFERED 325MG (EA)
	250257004270000	ASPIRIN SUPP 120MG
	250257004280000	ASPIRIN SUPP 200MG
	250257004290000	ASPIRIN SUPP 300MG
	250257004300000	ASPIRIN SUPP 600MG
	250257004310000	ASPIRIN TAB 325MG (EA)
	250257004320000	ASPIRIN TAB 325MG (2)
	250257004330000	ASPIRIN GUM 227.5MG (16)
	250257004340000	ASPIRIN TAB CHW 81MG (EA)
	250257004350000	ASPIRIN TAB CHW 81MG (2)
	250257004360000	ASPIRIN TAB SR 650MG
	250257004370000	ASPIRIN TAB EC 325MG
	250257004380000	ASPIRIN TAB EC 500MG
	250257004390000	ASPIRIN TAB EC 650MG
	250257004410000	ASPIRIN TAB EC 165MG
	250257004420000	ASPIRIN TAB EC 81MG
	250257004440000	ASPIRIN/CAFF, ANACIN TAB 400/32MG
	250257004500000	ASPIRIN/MAG/ALUM, ASCRIPTIN ES TAB 800/80/80MG
	250257004510000	ASPIRIN/MAG/ALUM, ASCRIPTIN TAB 325/50/50MG
	250257004250000	ASPIRIN TAB BUFFERED 325MG (2)
Ibuprofen	250250032790000	IBUPROFEN, MOTRIN TAB 300MG
	250250032800000	IBUPROFEN, MOTRIN TAB 400MG
	250250032810000	IBUPROFEN, MOTRIN TAB 600MG
	250250032820000	IBUPROFEN, MOTRIN TAB 800MG
	250250100580000	IBUPROFEN, MOTRIN DRPS 40MG/ML 15ML
	250250116820000	IBUPROFEN,CALDOLOR INJ 100MG/ML 4ML
	250250116830000	IBUPROFEN,CALDOLOR INJ 100MG/ML 8ML
	250250118790000	IBUPROFEN,CALDOLOR INJ 100MG
	250250130120000	IBUPROFEN, MOTRIN TAB CHW 100MG
	250257032770000	IBUPROFEN, ADVIL TAB 200MG
	250257032780000	IBUPROFEN, MOTRIN TAB 100MG
	250257032830000	IBUPROFEN, MOTRIN TAB CHW 50MG

	250257032840000	IBUPROFEN, PEDIAPROFEN SUSP 100MG/5ML 10ML
	250257032850000	IBUPROFEN, PEDIAPROFEN SUSP 100MG/5ML 120ML
	250257032860000	IBUPROFEN, PEDIAPROFEN SUSP 100MG/5ML 20ML
	250257032870000	IBUPROFEN, PEDIAPROFEN SUSP 100MG/5ML 30ML
	250257032880000	IBUPROFEN, PEDIAPROFEN SUSP 100MG/5ML 40ML
	250257032890000	IBUPROFEN, PEDIAPROFEN SUSP 100MG/5ML 5ML
	250257068010000	IBUPROFEN, PEDIAPROFEN SUSP 100MG/5ML 60ML
Naproxen	250250045850000	NAPROXEN NA, ANAPROX DS TAB 500(550)MG
	250250045860000	NAPROXEN NA, ANAPROX TAB 250(275)MG
	250250045870000	NAPROXEN, NAPROSYN SUSP 125MG/5ML 10ML
	250250045880000	NAPROXEN, NAPROSYN SUSP 125MG/5ML 30ML
	250250045890000	NAPROXEN, NAPROSYN SUSP 125MG/5ML 5ML
	250250045900000	NAPROXEN, NAPROSYN TAB 250MG
	250250045910000	NAPROXEN, NAPROSYN TAB 375MG
	250250045920000	NAPROXEN, NAPROSYN TAB 500MG
	250250046010000	NAPROXEN, NAPROSYN-EC TAB SR 375MG
	250250046020000	NAPROXEN, NAPROSYN-EC TAB SR 500MG
	250250111360000	NAPROXEN/ESOMEPRAZOLE, VIMOVO TAB DR 375/20MG
	250250111370000	NAPROXEN/ESOMEPRAZOLE, VIMOVO TAB DR 500/20MG
	250257045840000	NAPROXEN NA, ALEVE TAB 200(220)MG
Meloxicam	250250042990000	MELOXICAM, MOBIC TAB 7.5MG
Celecoxib	250250011910000	CELECOXIB, CELEBREX CAP 100MG
	250250011920000	CELECOXIB, CELEBREX CAP 200MG
	250250104570000	CELECOXIB, CELEBREX CAP 400MG
	250250114300000	CELECOXIB, CELEBREX CAP 50MG
Indomethacin	250250033320000	INDOMETHACIN, INDOCIN CAP 25MG
	250250033330000	INDOMETHACIN, INDOCIN CAP 50MG
	250250033340000	INDOMETHACIN, INDOCIN CAP SR 75MG
	250250033350000	INDOMETHACIN, INDOCIN SUPP 50MG
	250250033360000	INDOMETHACIN, INDOCIN SUSP 25MG/5ML 10ML
	250250033370000	INDOMETHACIN, INDOCIN SUSP 25MG/5ML 30ML
	250250033380000	INDOMETHACIN, INDOCIN SUSP 25MG/5ML 5ML
	250250033390000	INDOMETHACIN, INDOCIN VL 1MG
Toradol	250250036120000	KETOROLAC, ACULAR OP SOL 0.5% 3ML
	250250036130000	KETOROLAC, ACULAR OP SOL 0.5% 5ML
	250250036140000	KETOROLAC, TORADOL INJ 15MG/ML 1ML
	250250036150000	KETOROLAC, TORADOL INJ 30MG/ML 1ML
	250250036160000	KETOROLAC, TORADOL INJ 30MG/ML 2ML
	250250036170000	KETOROLAC, TORADOL TAB 10MG
Diclofenac	250250018340000	DICLOFENAC/MISOPROST, ARTHROTEC TAB 50MG/200MCG
	250250018350000	DICLOFENAC/MISOPROST, ARTHROTEC TAB 75MG/200MCG
	250250018400000	DICLOFENAC NA, VOLTAREN TAB DR 25MG
	250250018410000	DICLOFENAC NA, VOLTAREN TAB DR 50MG
	250250018420000	DICLOFENAC NA, VOLTAREN TAB DR 75MG
	250250018430000	DICLOFENAC POT, CATAFLAM TAB 50MG
	250250124500000	DICLOFENAC, DYLOJECT INJ 37.5MG/ML 1ML
	250250126350000	DICLOFENAC, DYLOJECT INJ 0.5MG

Table S2. Comorbidity prevalence in those with and without AKI, stroke, and GIB

	AKI									
Comorbidity	With AKI (n)	%	Without AKI (n)	%	OR	95% CI	p-value	aOR	95% CI	p-value

CHF	2,332	13.3	15,162	86.7	9.76	9.32-10.2	**<0.001**	2.77	2.63-2.92	**<0.001**
HIV/AIDS	19	0.11	17,475	99.9	3.68	2.32-5.83	**<0.001**	3.08	1.88-5.04	**<0.001**
Diabetes with complications	1,356	7.75	16,138	92.3	9.24	9.72-9.80	<0.001	2.02	1.89-2.16	<0.001
HTN	15,496	88.6	1,998	11.4	0.10	8.60-9.50	**<0.001**	5.27	5.02-5.53	**<0.001**
Hypothyroidism	3,131	17.9	14,363	82.1	1.80	1.75-1.90	**<0.001**	1.12	1.07-1.16	**<0.001**
Liver disease	332	1.90	17,162	98.1	3.30	2.90-3.60	**<0.001**	1.92	1.71-2.17	**<0.001**
Peripheral vascular disease	1,030	5.89	16,464	94.1	4.30	4.00-4.60	**<0.001**	1.32	1.23-1.42	**<0.001**
Pulmonary circulation disorder	765	4.37	16,729	95.6	7.80	7.20-8.40	**<0.001**	2.52	2.30-2.75	**<0.001**
Renal failure	6,222	35.6	11,272	64.4	24.8	23.9-25.6	**<0.001**	11.15	10.8-11.6	**<0.001**
Chronic PUD	6	0.03	17,488	99.07	2.80	1.20-6.30	0.100	-	-	-
Valvular disease	1,215	6.95	16,279	93.1	2.80	2.60-2.90	**<0.001**	1.07	1.00-1.15	**0.037**
Chronic pulmonary disease	4,046	23.1	13,448	76.9	2.60	2.60-2.70	**<0.001**	1.52	1.46-1.58	**<0.001**
	**Stroke**									
**Comorbidity**	**With Stroke (n)**	**%**	**Without Stroke (n)**	**%**	**OR**	**95% CI**	**p-value**	**aOR**	**95% CI**	**p-value**
CHF	0	0.00	475	100.0	-	-	0.579	2.16	1.70-2/74	**<0.001**
HIV/AIDS	89	8.81	26221	91.2	5.66	4.55-7.00	**<0.001**	-	-	-
Diabetes with complications	37	3.66	15244	96.3	3.80	2.80-5.40	**<0.001**	1.50	1.07-2.11	**0.020**

HTN	827	81.9	727839	18.1	5.20	4.40-6.10	**<0.001**	4.03	3.41-4.75	**<0.001**
Hypothyroidism	196	19.4	167914	80.6	2.00	1.70-2.30	**<0.001**	1.37	1.17-1.60	**<0.001**
Liver disease	8	0.79	9531	99.2	1.30	0.65-2.60	0.458	-	-	-
Peripheral vascular disease	79	7.82	232192	92.2	5.60	4.50-7.10	**<0.001**	2.62	2.06-3.33	**<0.001**
Pulmonary circulation disorder	46	4.55	9746	95.4	7.60	5.60-10.2	**<0.001**	2.70	1.97-3.72	**<0.001**
Renal failure	110	10.9	39798	89.1	4.70	3.80-5.70	**<0.001**	1.92	1.55-2.38	**<0.001**
Chronic PUD	0	0.00	197	100.0	-	-	0.721	-	-	-
Valvular disease	106	10.5	41914	89.5	4.30	3.50-5.20	**<0.001**	2.14	1.73-2.65	**<0.001**
Chronic pulmonary disease	188	18.6	161673	81.4	2.00	1.70-2.30	**<0.001**	1.24	1.05-1.45	**0.011**
	**GIB**									
**Comorbidity**	**With GIB (n)**	**%**	**Without GIB (n)**	**%**	**OR**	**95% CI**	**p-value**	**aOR**	**95% CI**	**p-value**
CHF	0	0.00	475	100.0	-	-	0.711	2.81	2.03-3.88	**<0.001**
HIV/AIDS	53	11.7	26257	88.3	7.80	5.90-10.4	**<0.001**	-	-	-
Diabetes with complications	13	2.88	15268	97.1	3.00	1.70-5.20	**<0.001**	0.97	0.55-1.72	0.926
HTN	320	70.8	728,346	29.2	2.80	2.30-3.40	**<0.001**	1.92	1.55-2.38	**<0.001**
Hypothyroidism	66	14.6	168044	85.4	1.40	1.10-2.80	**0.008**	1.01	0.77-1.31	0.945
Liver disease	13	2.88	9526	97.1	4.80	2.80-8.40	**<0.001**	3.22	1.85-5.61	**<0.001**
Peripheral vascular disease	40	8.85	23231	91.2	6.40	4.60-8.90	**<0.001**	2.88	2.04-4.05	**<0.001**

Pulmonary circulation disorder	27	5.97	9765	94.0	10.00	6.80-14.9	**<0.001**	3.29	2.15-5.05	**<0.001**
Renal failure	64	14.2	39844	85.8	6.30	4.80-8.20	**<0.001**	2.84	2.12-3.82	**<0.001**
Chronic PUD	0	0.00	197	100.0	-	-	0.811	-	-	-
Valvular disease	43	9.51	41977	90.5	4.30	3.50-5.20	**<0.001**	1.76	1.26-2.47	**0.001**
Chronic pulmonary disease	115	25.4	161746	74.6	2.00	1.70-2.30	**<0.001**	1.95	1.56-2.43	**<0.001**

Bolded values are statistically significant ($p < 0.05$).

Section S1. Criteria used to define non-elective cases for exclusion

- Fracture of the pelvis or lower limbs coded in ANY discharge diagnosis field
- A concurrent partial hip or knee arthroplasty procedure
- A concurrent revision, resurfacing, or implanted device/prosthesis removal procedure
- Mechanical complication coded in ANY discharge diagnosis field
- Malignant neoplasm of the pelvis, sacrum, coccyx, lower limbs, or bone/bone marrow or a disseminated malignant neoplasm coded in ANY discharge diagnosis field
